# Musculoskeletal Aging and Sarcopenia in the Elderly

**DOI:** 10.3390/ijms23052808

**Published:** 2022-03-04

**Authors:** Emanuele Marzetti

**Affiliations:** 1 Fondazione Policlinico Universitario “Agostino Gemelli” IRCCS, 00168 Rome, Italy; emanuele.marzetti@policlinicogemelli.it; Tel.: +39-(06)-3015-5559; 2 Department of Geriatrics and Orthopedics, Università Cattolica del Sacro Cuore, 00168 Rome, Italy

The loss of skeletal muscle mass and strength/function, referred to as sarcopenia, is a pervasive feature of aging [1]. The remarkable prevalence of sarcopenia and its association with a broad range of negative health-related outcomes have instigated a great deal of research on the pathophysiology of muscle aging. This has led to the identification of several biological pathways that may be exploited for biomarker discovery and/or therapeutic purposes. This Special Issue convened basic and clinical researchers working in the areas of sarcopenia and muscle physiology to foster our understanding of the molecular events associated with muscle aging and their modulation by specific interventions.

Omics platforms are especially well-suited for unveiling complex molecular patterns that might be dissected to discover specific biological pathways. The study by Zampino et al. [2] is a notable example of the application of such an approach to the study of muscle aging. Through the measurement of the plasma concentration of a large protein array, the authors identified a proteomic signature of muscle mitochondrial function assayed by phosphorous magnetic resonance spectroscopy [2]. The relevance of mitochondrial dysfunction to sarcopenia is further highlighted by the observation that alterations in intracellular calcium handling impact mitochondrial bioenergetics [3]. Muscle fiber denervation, an event involved in the pathogenesis of sarcopenia, induces damage of membrane structures involved in calcium handling and excitation–contraction coupling, and disruption of the mitochondrial network [3]. These alterations were also described in physically inactive old mice and were rescued by electrical stimulation regular exercise in both rodents and older persons [3].

The maintenance of a functional mitochondrial network in skeletal myofibers relies on the fine regulation of a set of mitochondrial quality control (MQC) pathways, involving mitochondrial proteostasis, dynamics, biogenesis, and mitochondrial autophagy (mitophagy) [4]. The efficiency of MQC declines with advancing age in various tissues, including muscle, which is considered to be a major factor in the development of sarcopenia [5]. Indeed, alterations in mitochondrial dynamics and declining mitochondrial turnover cause an accumulation of dysfunctional organelles within skeletal myofibers, leading to impaired bioenergetics and activation of catabolic pathways [6]. It is noteworthy that the impact of myocyte mitochondrial dysfunction is not limited to the muscle, but can extend to distant organs (e.g., liver, heart, pancreas, white adipose tissue) and affect whole-body metabolic homeostasis through the release into the bloodstream of myomitokines, chiefly fibroblast growth factor 21 and growth and differentiation factor 15 [6].

Similar to defective autophagy, an overactivation of this degradative pathway, for instance during starvation, leads to muscle tissue depletion [7]. Malnutrition is frequently observed in patients with congestive heart failure (CHF) and contributes to hyperactivation of autophagy in cardiac and skeletal myocytes, which is thought to play a prominent role in the progression of heart dysfunction and muscle atrophy [8]. Indeed, sarcopenia is highly prevalent in older adults with CHF [9] and independently predicts poor prognosis [10]. Supplementation with essential amino acids may promote heart and skeletal muscle anabolism and improve survival in patients with CHF, at least partly through favoring mitochondrial biogenesis and attenuating the overactivation of autophagy [11]. Hence, the nutritional status should be carefully monitored in patients with CHF, and essential amino acid supplementation should be considered to mitigate cardiac dysfunction and muscle atrophy resulting from a maladaptive overactivation of autophagy [8].

Taurine, a non-essential amino acid abundant in nuts, shellfish, eggs, meat, and dairy products, is another promising nutrient that might be supplemented to promote muscle health and counteract sarcopenia [12]. The administration of taurine to myogenic L6 cells was shown to stimulate cell differentiation by downregulating the expression inflammatory molecules and through modulating autophagy and apoptosis [12].

Together with optimal nutrition, physical activity and exercise are the most effective interventions to prevent and treat sarcopenia [13]. Physical exercise, besides promoting muscle hypertrophy and strength gain, is well-known for its beneficial effects on the cardiovascular system and whole-body metabolism [14]. These effects are conveyed, at least partly, through the release of myokines, such as decorin, insulin-like growth factor 1, myonectin, apelin, musclin, and interleukin 6 [15]. Interestingly, time-scheduled physical exercise has been shown to restore the circadian rhythm in the skeletal muscle [16], which is altered during aging and in people with shift work or sleep disorders. Disruption of the circadian rhythm has been associated with detrimental changes in body composition and increased risk of sarcopenia [17,18]. The mechanisms by which circadian rhythm disruption impacts muscle health are not fully elucidated. However, studies in mice lacking CLOCK and BMAL1 have shown that clock gene deficiency causes mitochondrial dysfunction and muscle degeneration [19]. Hence, the restoration of the circadian rhythm through time-scheduled exercise might amplify the beneficial effects of exercise on muscle in older adults with sarcopenia [20]. This possibility warrants further investigation.

## Data Availability

No data were generated for the present article.
